# Anoctamin-1 Cl^−^ channels in nociception: activation by an *N*-aroylaminothiazole and capsaicin and inhibition by T16A[inh]-A01

**DOI:** 10.1186/s12990-015-0061-y

**Published:** 2015-09-12

**Authors:** Farah Deba, Bret F. Bessac

**Affiliations:** Department of Pharmaceutical Sciences, I. L. Rangel College of Pharmacy, Texas A&M Health Science Center, 1010 West Avenue B MSC 131, Kingsville, TX 78363 USA

**Keywords:** Anoctamin, ANO1, TMEM16A, T16Ainh-A01, Aroylaminothiazole, Nociception, DRG neuron, TRPV1, Pain

## Abstract

**Background:**

Anoctamin 1 (ANO1 or TMEM16A) Ca^2+^-gated Cl^−^ channels of nociceptor neurons are emerging as important molecular components of peripheral pain transduction. At physiological intracellular Cl^−^ concentrations ([Cl^−^]_i_) sensory neuronal Cl^−^ channels are excitatory. The ability of sensory neuronal ANO1 to trigger action potentials and subsequent nocifensive (pain) responses were examined by direct activation with an *N*-aroylaminothiazole. ANO1 channels are also activated by intracellular Ca^2+^ ([Ca^2+^]_i_) from sensory neuronal TRPV1 (transient-receptor-potential vallinoid 1) ion channels and other noxicant receptors. Thus, sensory neuronal ANO1 can facilitate TRPV1 triggering of action potentials, resulting in enhanced nociception. This was investigated by reducing ANO1 facilitation of TRPV1 effects with: (1) T16A[inh]-A01 ANO1-inhibitor reagent at physiological [Cl^−^]_i_ and (2) by lowering sensory neuronal [Cl^−^]_i_ to switch ANO1 to be inhibitory.

**Results:**

ANO1 effects on action potential firing of mouse dorsal root ganglia (DRG) neurons in vitro and mouse nocifensive behaviors in vivo were examined with an *N*-aroylaminothiazole ANO1-activator (E-act), a TRPV1-activator (capsaicin) and an ANO1-inhibitor (T16A[inh]-A01). At physiological [Cl^−^]_i_ (40 mM), E-act (10 µM) increased current sizes (in voltage-clamp) and action potential firing (in current-clamp) recorded in DRG neurons using whole-cell electrophysiology. To not disrupt TRPV1 carried-Ca^2+^ activation of ANO1 in DRG neurons, ANO1 modulation of capsaicin-induced action potentials was measured by perforated-patch (Amphotericin–B) current-clamp technique. Subsequently, at physiological [Cl^−^]_i_, capsaicin (15 µM)-induced action potential firing was diminished by co-application with T16A[inh]-A01 (20 µM). Under conditions of low [Cl^−^]_i_ (10 mM), ANO1 actions were reversed. Specifically, E-act did not trigger action potentials; however, capsaicin-induced action potential firing was inhibited by co-application of E-act, but was unaffected by co-application of T16A[inh]-A01. Nocifensive responses of mice hind paws were dramatically induced by subcutaneous injections of E-act (5 mM) or capsaicin (50 µM). The nocifensive responses were attenuated by co-injection with T16A[inh]-A01 (1.3 mM).

**Conclusions:**

An ANO1-activator (E-act) induced [Cl^−^]_i_-dependent sensory neuronal action potentials and mouse nocifensive behaviors; thus, direct ANO1 activation can induce pain perception. ANO1-inhibition attenuated capsaicin-triggering of action potentials and capsaicin-induced nocifensive behaviors. These results indicate ANO1 channels are involved with TRPV1 actions in sensory neurons and inhibition of ANO1 could be a novel means of inducing analgesia.

**Electronic supplementary material:**

The online version of this article (doi:10.1186/s12990-015-0061-y) contains supplementary material, which is available to authorized users.

## Background

Acute peripheral pain is an essential sensation evolved to deter exposure of normal tissues to harmful environments and to prevent further injury of damaged and inflamed tissues. Peripheral neuropathies and inflammatory conditions can heighten pain perception to noxious stimuli (hyperalgesia) and change the perception of non-noxious stimuli to be painful (allodynia). For millions of people, the hyperalgesia and allodynia occurs chronically and becomes intolerable and debilitating. Some common examples are back injuries, diabetic neuropathy, arthritis, dermatitis, oral and ocular diseases [1, 2]. Chronic peripheral pain not only alters the quality of life, but also costs billions of dollars due to the loss of work productivity and the health care to alleviate and manage the pain, including chronic medications [2, 3]. Great strides have been made to improve pain management. However, the physiological and pathophysiological molecular components of peripheral pain, hyperalgesia, allodynia and the progression from acute to chronic pain, as well as the effects of targeting these sites with pharmaceuticals, are not yet fully understood.

Acute peripheral pain transduction is mediated by unmyelinated C-fiber and thinly myelinated Aδ fiber nociceptive neurons. The peripheral terminals of these somatosensory neurons express specific receptors for: (1) environmental hazards (extreme temperatures, reactive chemicals…) and (2) signaling factors released by damaged and inflamed tissues. Activation of the receptors evokes a graded depolarizing potential and often an increase of intracellular Ca^2+^, [Ca^2+^]_i_. If depolarization reaches levels to activate voltage-gated Na^+^ channels, then action potentials (APs) propagate along the processes to central terminal glutaminergic/peptidergic synapses. Subsequent activation of spinal/brainstem neurons elicits reflexes and transmits signals via lateral spinothalamic and related tracts to brain neurons. The brain filters and interprets the signals and executes the perception of pain and other protective responses [4]. Nociceptive neurons with injuries or innervating continuously inflamed tissues can become hypersensitivity to endogenous factors and environmental hazards. The pathophysiological change in sensory neurons contribute significantly to evoking hyperalgesia and allodynia [5]. Central nervous system neurons are also involved in the perception of hyperalgesia and allodynia.

Recently, sensory neuronal Anoctamin 1 (ANO1) Ca^2+^-gated Cl^−^ channels have emerged as an important molecular component of nociception transduction in response to hazardous environmental stimuli and inflammatory signals. ANO1 channels also contribute to the related phenomena of hyperalgesia and allodynia [6–9]. Insights into sensory neuronal ANO1 contribution to nociception have emerged from its thermo-sensor properties and activation by increased [Ca^2+^]_i_ induced by inflammatory signaling factors and noxious environmental stimuli, as well as, the physiological functions of ANO1 in other tissues. However, the precise mechanisms of nociceptive function of ANO1 channels in sensory neurons requires further study.

ANO1 (also known as TMEM16A, DOG-1, ORAOV2 or TAOS-2) are channel proteins putatively formed of eight transmembrane spans arranged in a dimer [10–13]. In addition to somatosensory neurons, ANO1 channels are expressed in a variety of excitatory, secretory and other cell types [14–16]. ANO1 channels carry outward rectifying anion currents gated by heat and [Ca^2+^]_i_ (with KD_50_ in the submicromolar range), and pharmacological agents [17, 18]. Due to their high cytosolic Cl^−^ concentrations, [Cl^−^]_i_ and consequential Cl^−^ electrochemical equilibrium (E_Cl−_), sensory neuron Cl^−^ channels are excitatory; in contrast, central neuronal Cl^−^ channels are inhibitory [18]. Therefore, sensory neuronal ANO1 channels are presumed to participate as depolarizing Cl^−^ effluxes at resting potentials that can contribute to triggering APs [9].

ANO1 mediates the AP propagation and subsequent pain-like nociception to proteases released and activated by damaged and inflamed tissues; M-type K^+^ channels and transient-receptor-potential (TRP) cation channels are also involved. The proteases and their products, such as bradykinin (BK), are detected by sensory neuronal G-protein coupled receptors (GPCRs). Specifically, BK GPCR activation results in inositol-triphosphate (IP_3_) gating of IP_3_-receptor channels to release Ca^2+^ from intracellular stores. In sensory neurons, IP_3_-receptors are coupled with ANO1, thereby they directly delivering a large localized concentration of Ca^2+^ to activate ANO1 channels [6, 7]. The ensuing ANO1 depolarizing Cl^−^ fluxes then trigger APs that result in pain perception to the site of injury/inflammation. PAR2 (proteinase activated receptor-2) appears to activate nociceptive neuronal ANO1 by a similar mechanism [7].

ANO1, TRP channels, and other channels expressed on nociceptive neurons, detect elevated environmental temperatures and respond by membrane depolarization [20–23]. These specialized channels combine with the other thermosensitive channels to code the magnitude of heat intensity that results in the spectrum of psychophysical perception of temperature from comfortably warm to painfully hot [8, 24]. The best characterized of the thermosensitive channels are TRPV1 (TRP vallinoid 1) channels. Mammalian TRPV1 channels are responsible for the ‘hot’ sensation of capsaicin, the pungent ingredient of chili peppers; TRPV1 are also involved in general nociception, hyperalgesia and pro-inflammatory neuropeptide release [24–27]. TRPV1 channels carry cations including Ca^2+^ that depolarize neurons [28]. TRPV1 and ANO1 are highly expressed and co-localized in nociceptive neurons [24, 30]. Recently, it has been shown that sensory neuronal TPRV1 and ANO1 channels interact. Thus, TRPV1-carried [Ca^2+^]_i_ can gate ANO1 in a manner similar to [Ca^2+^]_i_ induced by BK [31–34]. At physiological [Cl^−^]_i_, TRPV1-carried Ca^2+^ gating of ANO1 channels result in depolarizing Cl^−^ effluxes and combining with TRPV1 carried cation influxes to facilitate the triggering of APs. Thereby, pain perception and associated nocifensive behaviors to TRPV1 stimulation are intensified. This could be especially pronounced in heat detection, where ANO1 would be directly activated by heat and in direct activation by [Ca^2+^]_i_ from TRPV1, which is also activated by heat [23]. Contrariwise, sensory neuronal APs and painful responses by TRPV1 stimulation could be either diminished by blocking ANO1 channels or by lowering sensory neuronal [Cl^−^]_i_ enough to switch ANO1-carried Cl^−^ fluxes to be inhibitory.

Our research examined ANO1 involvement in nociception by: (1) manipulating [Cl^−^]_i_ and (2) using pharmacological tools to activate or inhibit ANO1 channels activity. We used an N-aroylaminothiazole ANO1-activator reagent, E-act, to induce ANO1-like electrophysiological currents in sensory neurons and evoke [Cl^−^]_i_–dependent action potentials that were independent of heat or [Ca^2+^]_i_. Furthermore, subcutaneous applications of E-act into mouse paws, in vivo, induced nocifensive behaviors. To the best of our knowledge, these studies are the first to demonstrate that direct activation of ANO1 channels by a pharmacological reagent results in sensory neuron APs and pain-associated nociceptive behaviors. This shows ANO1 activation alone is sufficient to trigger APs and implies direct activation of ANO1 can produce pain. Thus, ANO1 activation could be involved in facilitating the effects of many noxicants and a variety of pain disorders.

Pharmacological inhibitors of ANO1 have been examined in sensory neurons to diminish BK effects [6]. Our research similarly used inhibitors of ANO1 to diminish: 1) the in vitro effects of TRPV1 activation by capsaicin on sensory neuronal AP firing, and 2) in vivo mouse nocifensive behavioral responses [25–28]. We report here that co-application with ANO1-inhibitor, T16A[inh]-A01 (ANO1-inh), attenuated capsaicin-induced sensory neuronal AP firing at physiological [Cl^−^]_i_. At low [Cl^−^]_i_, ANO1 channels are inhibitory. Therefore, ANO1-inh inhibitory effects were absent and instead direct activation of ANO1 by E-act diminished capsaicin-induced APs. Subcutaneous co-applications of ANO1-inh diminished mouse nocifensive behavioral responses to capsaicin [34]. These results indicate: (1) ANO1 Cl^−^ channels modulate TRPV1 channels effects and (2) the potential of specific pharmaceutical inhibitors of ANO1 as local anesthetics.

## Results

### E-act activates and T16A[inh]-A01 inhibits ANO1 in transfected HEK-293 cells

**Fig. 1:**
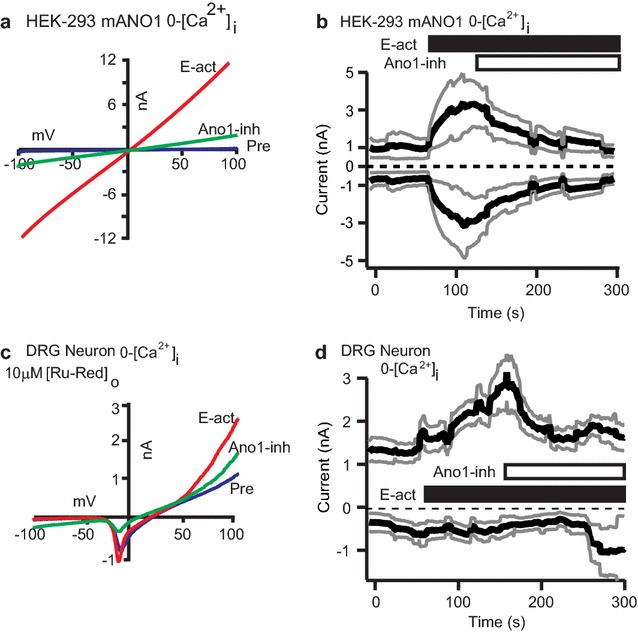
ANO1 currents are induced by ANO1-activator and diminished by ANO1-inhibitor in mouse DRG neurons and transfected HEK-293 cells. **a** Traces of currents recorded to voltages ramped from −100 to +100 mV of a representative mANO1 transfected HEK-293t cell (mANO1-HEK293): before perfusion (*blue trace* Pre) and following bath perfusions of *N*-aroylaminothiazole (E-act, 10 µM, *red trace*) activating ANO1, and sequential inhibition of E-act effects by mixing in ANO1-inhibitor T16A[inh]-A01 (ANO1-inh, 20 µM, *green trace*). Recording pipettes had 0-Ca^2+^; V_H_ = 0 mV. **b** Averaged current sizes recorded at −80 mV and +80 mV (*black lines*), increased following 10 µM E-act (*black bar* at 70 s) and decreased by ANO1-inh with E-act (*white bar* at 140 s) bath perfusions for mANO1-HEK293 cells (n = 3). *Gray lines* represent SE. **c** Traces of ANO1-like outwardly rectifying currents recorded to voltages ramped from −100 to +100 mV of a representative DRG neuron: before activation (*blue trace* Pre), activation by 10 µM E-act (*red trace*) bath perfusion, and inhibition by a perfusion mixture of E-act with 20 µM ANO1-inh (*green trace*). **d** Average current sizes recorded at −80 mV and +80 mV (*black lines*), following 10 µM E-act (*black bar* at 80 s) perfusion and a sequential perfusion mixture of 20 µM ANO1-inh with E-act (*white bar* at 180 s) for DRG neurons (n = 7). *Gray lines* represent SE. In DRG neurons (**c**, **d**), all bath and applications contained 10 µM ruthenium red to block TRP channels and recording pipette contained 0-Ca^2+^. V_H_ = −70 mV. All intracellular solutions were CsCl-based. Records of currents were made over 200 ms at 1 Hz intervals in the whole-cell configuration

Our studies examined ANO1 Cl^−^ channels’ function in dorsal root ganglia (DRG) neurons and their role in mouse nociception/pain. Pivotal to these studies were pharmacological manipulation of ANO1 channels expressed in DRG neurons by an *N*-aroylaminothiazole ANO1-activator, E-act, and ANO1-inhibitor, T16A[inh]-A01 (ANO1-inh). Namkung et al., 2011, originally discovered and characterized E-act’s ability to activate ANO1 by: (1) Cl^−^ (I^−^) imaging and electrophysiology of salivary and airway cells and recombinant ANO1-expressing cells and (2) measuring bronchi submucosal glands stimulation and intestinal smooth muscle contractions [18]. ANO1-inh was also discovered by the same laboratory group using similar methods [35]. Following their discovery, ANO1-inh has been a powerful tool for inhibiting ANO1 channels in studies of the epithelia and the vasculature as well as bradykinin actions in sensory neurons [7, 36–38]. E-act (10 µM) activation of ANO1 and ANO1-inh (20 µM) inhibition of E-act effects were verified with whole-cell patch-clamp electrophysiology of recombinant mouse ANO1 (mANO1) transfected in HEK-293t cells. Currents were recorded to voltage ramps of −100 to +100 mV over 200 ms at every second; in between ramps, potential was held (V_H_) to 0 mV. E-act application induced large sigmoidal or linear current–voltage curves (Fig. 1a). Therefore, inward currents at −80 mV and outward currents at +80 mV were on average equal sizes (Fig. 1b). The linear current–voltage curves were similar for mANO1 current–voltage curves activated by high [Ca^2+^]_i_ (10 µM or more) and were also inhibited by 20 µM ANO1-inh [39]. Individual mANO1 inward current sizes (at −80 mV) and outward current sizes (at +80 mV) that were recorded at 10 µM-[Ca^2+^]_i_ are displayed (n = 4) in Additional file 1: Figure S1. mANO1 currents inactivated spontaneously in several recordings and were excluded in our studies. It is possible ANO1-inh effects on ANO1 currents at 10 µM-[Ca^2+^]_i_ are skewed by channel inactivation.

### E-act activates and T16A[inh]-A01 inhibits ANO1 in DRG neurons

ANO1 expression in DRG neurons has been well established [24, 40]. Inward currents at −60 mV in DRG neurons have been deduced to be ANO1 currents. The currents are increased by [Ca^2+^]_i_ from activation of BK-GPCR or TRPV1 and then inhibited by ANO1-inh or other Cl^−^ channel inhibitors [7, 32]. To further understand ANO1 channels physiology in DRG neurons, we recorded whole-cell currents to voltage ramps from −100 to +100 mV in mouse primary cultured DRG neurons in response to direct activation of ANO1 by E-act. E-act (10 µM) perfusion induced outward rectifying current–voltage curves that were subsequently inhibited by co-application with 20 µM ANO1-inh; 12 out of 18 DRG neurons tested showed E-act induced currents, however, only 7 of these patches were used with ANO1-inh (Fig. 1c). As shown in Fig. 1d, the average inward currents (at −80 mV) induced by E-act were minimal, while relatively large average outward currents (at +80 mV) occurred. Currents were recorded every second for 200 ms with V_H_ = −70 mV. Recording-pipette solutions contained Cs^+^ to block K^+^ channels and extracellular solutions contained ruthenium red (10 µM) to block TRP and other divalent cation channels.

The lack of large inward currents was surprising considering that: (1) receptor-mediated [Ca^2+^]_i_ activation of ANO1 in DRG neurons induced large (>400 pA) inward currents at −60 mV and (2) our recordings of E-act inducing large inward currents (linear current–voltage curves) for recombinant ANO1 [7, 32]. However, recordings of native ANO1 reported in other tissues are very similar to E-act-induced outward rectifying currents in DRG neurons [6, 33]. Moreover, recombinant ANO1 current–voltage curves induced by 1 µM or less [Ca^2+^]_i_ have similar outward rectification as native ANO1 [38]. E-act induced DRG currents being attributed to ANO1 channels activation and not to off target effects were supported by: (1) E-act induction of currents in recombinant ANO1 expressing cells and (2) co-application of ANO1-inh reduction of the E-act induced currents in DRG neurons. ANO1-inh does not have actions on sensory neuronal voltage-gated Na^+^, Ca^2+^ or K^+^ channels [32]. In certain experiments, V_H_ was switched from −70 to 0 mV (and vice versa) to assure that: (1) outward rectification was not due to voltage regulation of ANO1 and (2) E-act or ANO1-inh did not interfere with voltage-gated Na^+^ channels (V_H_ = 0 mV closes certain, albeit not all, voltage-gated Na^+^ channels by the inactivation h-gate). Under these conditions, there was no noticeable effect (data not shown).

### E-act evokes action potentials in DRG neurons dependent on [Cl^−^]_i_

**Fig. 2:**
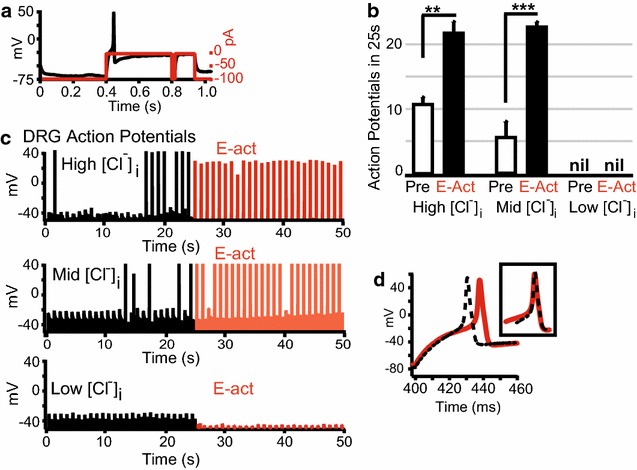
ANO1-activator evokes action potentials in DRG neurons that are dependent on intracellular Cl^−^. **a** Membrane potential (V_m_) trace (*black trace*) adjusted by current injections (*red trace*) of a representative DRG neuron are shown to illustrate the protocol of whole-cell current-clamp recordings. The current injections necessary to lower V_m_ near to −70 mV varied; therefore, they were determined empirically at the start of current clamp. **b** Average number of action potentials (APs) by DRG neurons over 25 s were significantly increased by ANO1 activation from an *N*-aroylaminothiazole (E-act, 10 µM, *black bars*) perfusion compared to prior to application (Pre *white bars*); when intracellular solution concentrations were 160 mM Cl^−^ (high [Cl^−^]_i_, n = 3) or 40 mM Cl^−^ (mid [Cl^−^]_i_, n = 3), but not 10 mM Cl^−^ (low [Cl^−^]_i_, n = 3). *Error bars* are SE. (***p > 0.001; **p > 0.01). **c** APs recorded in representative DRG neurons before application (*black* Pre) and following perfusion of E-act (*red*) with intracellular solutions of High [Cl^−^]_i_ (upper graph), Mid [Cl^−^]_i_ (*middle graph*) or Low [Cl^−^]_i_ (*bottom graph*). **d** A representative DRG neuron voltage–time plot of: AP spontaneously occurring before application (*broken line*), and AP induced by E-act (*solid line*). (*Insert box*) APs superimposed upon each other to demonstrate that there was no difference in the shape of the voltage–time plots, only the time of action potential onset

Sensory neurons have relatively high (~40 mM) intracellular Cl^−^, [Cl^−^]_i_, thus the Cl^−^ electrochemical equilibrium (E_Cl-_) is approximately −30 mV [19]. This is near the voltage required to activate voltage-gated Na^+^ channels responsible for action potential (AP) propagation. We examined if at high [Cl^−^]_i_, (160 mM; E_Cl−_ = 1.1 mV) or physiological/mid [Cl^−^]_i_ (40 mM; E_Cl_^−^ = −34 mV), activation of ANO1 channels would trigger APs in sensory neurons. While, at low [Cl^−^]_i_ (10 mM, E_Cl−_ = −69 mV), ANO1 activation would inhibit AP firing. Whole-cell current clamp electrophysiology of primary cultured DRG neuronal membrane potential (V_m_) was used to record APs (V_m_ spikes above 10 mV were considered APs). Currents were injected to adjust the non-excited V_m_ to −30 ± 10 mV, a level slightly below, the voltage necessary to activate voltage-gated channels. Voltage-gated channels responsible for APs were then reset by current injections to bring V_m_ to −70 ± 10 mV [40] (Fig. 2a).

Direct activation of sensory neuronal ANO1 by E-act (10 µM) bath perfusion significantly increased APs from baseline when [Cl^−^]_i_ was at high [Cl^−^]_i_ (n = 3) or mid [Cl^−^]_i_ (n = 3). The average APs for 25 s occurring at baseline and after E-act are presented in Fig. 2b. {*Average APs for: High [Cl*^*−*^*]*_*i*_*at baseline* = *10.3* ± *1.4 and E*-*act* = *21.7* ± *2.0; Mid [Cl*^*−*^*]*_*i*_*at baseline* = *5.0* ± *1.4 and E*-*act* = *23.3* ± *2.0}.* There were no E-act induced APs in DRG neurons with Low [Cl^−^]_i_ (n = 3). AP firing at baseline and during E-act (10 µM) perfusion are illustrated in V_m_-time plots for represented DRG neurons with intracellular solutions of: high [Cl^−^]_i_, mid [Cl^−^]_i_ and low [Cl^−^]_i_ (Fig. 2c). In these graphs, V_m_ recordings are shown above ˗30 ± 10 mV (voltages below ˗40 mV occurred but are not shown). E-act-induced AP firings of DRG neurons were independent of other known ANO1 activators: [Ca^2+^]_i_ and temperatures above 27 °C (Recording electrode solutions were devoid of Ca^2+^ and recordings occurred at room temperature, ~22 °C).

ANO1 outward rectifying currents in Fig. 1b, c, would imply that ANO1 would carry large influxes of Cl^−^ at the positive potentials of APs. This could result in ANO1 channel activation enhancing the repolarization phase of APs. However, when we compared individual APs spontaneously occurring at baseline to APs induced by E-act application in the same DRG neurons, the APs were similar in their depolarization and repolarization phases. This is illustrated in the insert of Fig. 2d with the spontaneous baseline AP superimposed by the E-act induced AP. We did observe a delay of the initiation of APs induced by E-act, as the two APs indicate in the representative DRG neuron of Fig. 2d. The delay between the APs was statistically significant for DRG neurons with physiological [Cl^−^]_i_. However, V_m_ at the repolarization/resting potential phase of the current pulse also often decreased by 10-20 mV over the time course of the experiment and could explain these results. {*Average time of AP peak voltage occurred at: baseline* = *437.6* ± *1.4* *ms, n* = *15; E*-*act* = *443.9* ± *0.7* *ms, n* = *40; p* < *0.001}*.

### E-act induces nocifensive behaviors

**Fig. 3:**
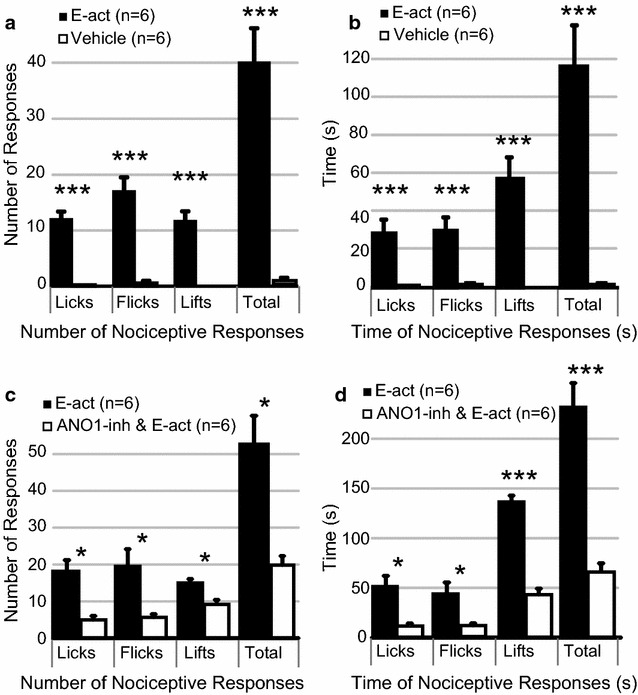
Mouse nocifensive behaviors are induced by ANO1-activator and attenuated by ANO1-inhibitor. The average *number* **a** and time spent in **b** nocifensive behavior responses (licks, flicks, lifts and total responses) of mice hind paws (n = 6) were significantly greater following subplantar injections of an *N*-aroylaminothiazole ANO1-activator (E-act, 5 mM, *black bar*) than vehicle (*white bar*) injections. The average *number* **c** and time spent in **d** nocifensive responses of mice hind paws (n = 6) to co-injections of 5 mM E-act with 1.3 mM of ANO1-inhibitor (ANO1-inh&E-act, *white bar*) were significantly fewer than subplantar injections of 5 mM E-act alone (*black bar*). All nocifensive behaviors were quantified from videos of the 5 min immediately following subplantar injections. Injection regimens occurred on different days and opposite paws of the same mice. *Error bars* are SE. (***p > 0.001, **p > 0.01, *p > 0.05)

APs in nociceptor neurons result in the synaptic release of glutamate and neuropeptides that activate spinal/brainstem neurons to transmit signals for pain perception by the brain. Our findings that E-act triggers APs in vitro could, thus, translate to E-act inducing peripheral pain-associated nocifensive behavioral responses in a mouse model in vivo. Therefore, nocifensive responses to paw injections (licks, flicks and lifts) were video recorded over 5 min. A single behavior, such as paw lift, could be maintained for a long time (over 1 min). Hence, not only the number of behavioral responses, but also the lengths of time for the responses were quantified. Immediately following subplantar 25 μl injections of E-act (5 mM) into the hind paw, mice displayed vigorous nocifensive responses that were significantly more in total number and time spent performing than the nonexistent effects of vehicle injections in the contralateral paw (Fig. 3a, b). *{Average total behaviors induced by: E*-*act (number* = *39.7* ± *6.5 and time spent* = *117.2* ± *20.6); vehicle (number* = *0.7* ± *0.3 and time spent* = *0.6* ± *1.5); n* = *6}.* Nocifensive responses to 5 mM E-act were significantly reduced when co-injected with 1.3 mM ANO1-inh (Fig. 3c, d). *{Average total behaviors induced by: E*-*act (number* = *52.7* ± *7.7 and time spent* = *231.7* ± *24); ANO1*-*inh with E*-*act (number* = *19.8* ± *2.5 and time spent* = *66* ± *8.7; n* = *6}.* The data suggest ANO1 activation by E-act induces peripheral pain that can be attenuated by ANO1-inh.

### T16A[inh]-A01 attenuates capsaicin evoked firing of action potentials by DRG neurons

ANO1 are Ca^2+^-gated Cl^−^ channels found in a variety of tissues, and many of ANO1 physiological functions involve gating by intracellular Ca^2+^ [14]. In sensory neurons, ANO1 gating by [Ca^2+^]_i_ (via IP_3_ receptor coupling) mediates bradykinin (BK) B_2_ receptor triggering of APs (K^+^ channel inhibition, and TRP channels are also involved) [6]. Lowering [Cl^−^]_i_ to shift E_Cl-_ to be more negative, renders ANO1 to be inhibitory and thereby suppresses bradykinin triggering of APs [6]. Bradykinin-evoked APs of sensory neurons, mediated by ANO1, are also suppressed by ANO1-inh or non-specific Cl^−^ channel blockers. (This was verified in our lab; data not shown).

Activation of other sensory neurons receptors, such as TRPV1, increase [Ca^2+^]_i_ that could gate ANO1. Heat, acidity, and capsaicin activate sensory neuronal TRPV1 channels to carry an influx of cations including Ca^2+^ that can trigger APs. ANO1 and TRPV1 directly interact and are co-expressed in sensory neurons [24, 32]. We propose that at high or physiological [Cl^−^]_i_, TRPV1-carried [Ca^2+^]_i_ activates ANO1. Subsequently, ANO1-carried Cl^−^ efflux positively feeds back to increase depolarization and thus increase the triggering of APs. ANO1-inhibitors would block ANO1 channels’ contribution to depolarization thereby diminishing TRPV1-triggered APs. Conversely, at low [Cl^−^]_i_, activation of ANO1 would inhibit TRPV1; moreover, ANO1-inhibitors would have little influence.

**Fig. 4:**
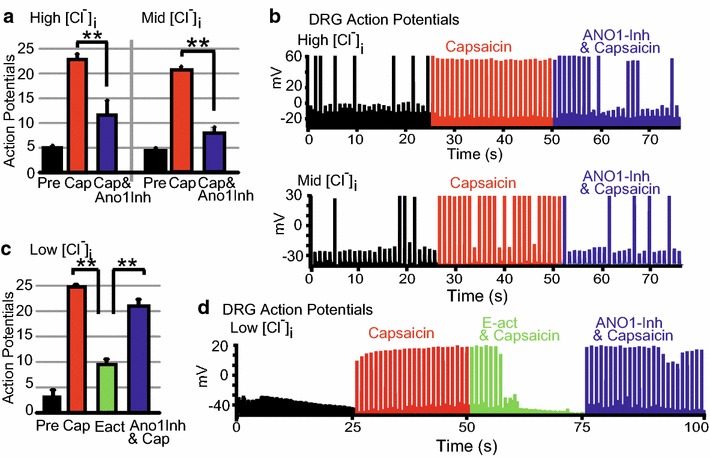
ANO1 modulation of capsaicin triggered action potentials in DRG neurons is determined by [Cl^−^]_i_. **a** The average number of action potentials (APs) by DRG neurons over 25 s increased by perfusion of TRPV1-activator capsaicin (15 µM, Cap, *red bars*) from baseline (Pre, *black bars*) were significantly lowered by perfusion of an ANO1-inhibitor 20 µM T16A[inh]-A01 mixed with capsaicin (Cap&ANO1-inh, *blue bars*) when the intracellular solutions contained concentrations of: 160 mM Cl^−^ (High [Cl^−^]_i_, n = 5) or 40 mM Cl^−^ (Mid [Cl^−^]_i_, n = 3). **b** APs recorded in representative DRG neurons with intracellular solutions of High [Cl^−^]_i_ (*upper graph*) or Mid [Cl^−^]_i_ (*lower graph*) at baseline (*black traces*) and following perfusions of 15 µM capsaicin (*red traces*) and 20 µM ANO1-inh/15 µM capsaicin mixture (*blue traces*). **c** In DRG neurons with intracellular solutions containing 10 mM Cl^−^ (Low [Cl^−^]_i_, n = 4), the increased average number of action potentials over 25 s by capsaicin (15 µM, *white bars*) perfusion from baseline (*black bars*) were significantly lowered by perfusion of an *N*-aroylaminothiazole ANO1-activator (E-act, 10 µM) mixed with 15 µM capsaicin (*green bars*). Perfusion of 20 µM ANO1-inh mixed with 15 µM capsaicin (*blue bars*) restored the action potential firing to the level of capsaicin alone. **d** APs recorded in a representative DRG neuron with intracellular solution of Low [Cl^−^]_i_ at baseline (*black traces*) and bath perfusions of 15 µM capsaicin (red traces), 10 µM E-act with capsaicin mixture (*green traces*) and 20 µM ANO1-inh with capsaicin (*blue traces*). *Error bars* are SE. (**p > 0.01). DRG neurons were recorded in perforated-patch current clamp with a similar current injection protocol as Fig. 2a

ANO1 modulation of capsaicin-induced action potentials in DRG neurons was measured by Ca^2+^ impermeable perforated-patch (Amphotericin-B) current-clamp technique to not disrupt TRPV1 carried-Ca^2+^ activation of ANO1. Currents were injected as described in the methods and shown in Fig. 2a. In our hands, it was not possible to determine ANO1 and TRPV1 co-activation using voltage-clamp because TRPV1 currents mask ANO1 current–voltage curves. Therefore, we examined if inhibition of ANO1 channels would remove the facilitation, and thus, lower TRPV1-mediated AP firing in DRG neurons. Perfusion of a saturating dose of capsaicin (15 µM) evoked robust DRG neuron AP firing that was significantly reduced by ANO1-inh (20 µM) mixed with capsaicin. This observation occurred when DRG neuron [Cl^−^]_i_ was altered to 160 mM (High [Cl^−^]_i_) or 40 mM (Mid [Cl^−^]_i_) by the recording-pipette milieu (Fig. 4a). *{Average APs for: High [Cl*^*−*^*]*_*i*_*at baseline* = *5* ± *0.4, capsaicin* = *22.8* ± *1.1 and ANO1*-*inh & capsaicin* = *11.6* ± *3.0, n* = *5; Mid [Cl*^*−*^*]*_*i*_*at baseline* = *4.3* ± *0.3, capsaicin* = *20.6* ± *0.7 and ANO1*-*inh & capsaicin* = *8.0* ± *1.2, n* = *3}.* APs over the time course of the experiment are shown in representative DRG neurons with High [Cl^−^]_i_ and Mid [Cl^−^]_i_ (Fig. 4b). Although, the results were similar to a simultaneous publication; a perplexing difference was that they recorded a series of APs induced by capsaicin with current injection continuously held near 0-pA for more than 10-s [32]. In our study, only one AP was recorded for a 0-pA current injection segment that lasted 0.12 or 0.4 s.

We then explored ANO1 modulation of capsaicin-induced APs in DRG neurons with Low [Cl^−^]_i_ (10 mM). At Low [Cl^−^]_i_, capsaicin (15 µM) still induced pronounced DRG neuron AP firing, which was significantly suppressed by co-application of ANO1-activator E-act (10 µM). Subsequently, co-application of ANO1-inh (20 µM) with capsaicin rescued AP firing back to levels of capsaicin alone (Fig. 4c). In Fig. 4d, APs over the time course of the experiment are shown in a representative DRG neuron with Low [Cl^−^]_i_. AP firing induced by capsaicin and induced by capsaicin with ANO1-inh were not significantly different from each other. They were significantly greater than the number of APs that occurred at baseline and with co-application of capsaicin with E-act. {*Average APs for: baseline* = *3.3* ± *1.3; capsaicin* = *24.8* ± *0.3; E*-*act & capsaicin* = *9.5* ± *1.0; ANO1*-*inh & capsaicin* = *21.0* ± *1.3; n* = *4}*. AP firing frequency induced by capsaicin was not significantly reduced in experiments with a sequential second perfusion of capsaicin (data not shown).

### Capsaicin-induced nocifensive behaviors are attenuated by T16A[inh]-A01

**Fig. 5:**
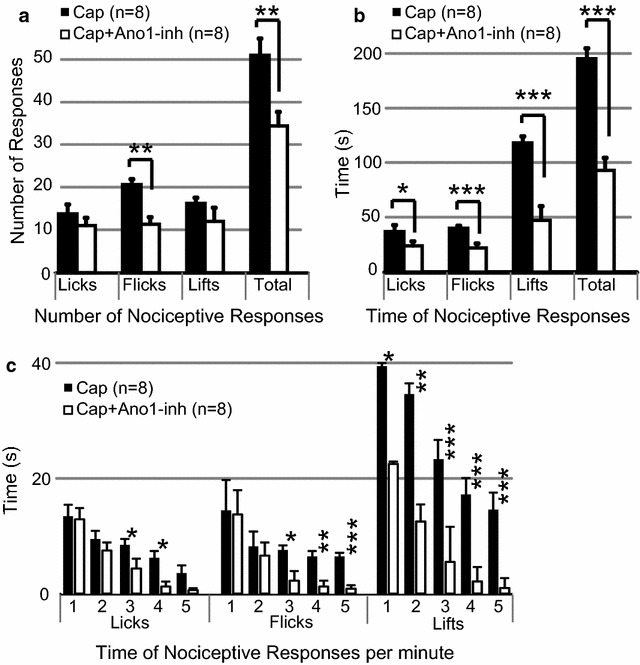
ANO1-inhibitor attenuates mouse nocifensive behavior responses to capsaicin. The average *number* (**a**) and *time spent in* (**b**) nocifensive behavior responses (licks, flicks, lifts and total responses) by mice hind paws (n = 8) to subplantar injections of 50 µM capsaicin (Cap *black bar*) were significantly more than subplantar injections of an Ano1-inhibitor 1.3 mM T16A[inh]-A01 mixed with 50 µM capsaicin (Cap + Ano1-inh, white bar) in contralateral paws, (pre-treated injection was given 2 h earlier with 1.3 mM Ano1-inh). **c** Average total *time* spent performing nocifensive behaviors at each minute increment (1-hrough 5th minute following injection of capsaicin (Cap *black bar*) or Ano1-inh mixed with capsaicin (Cap + Ano1-inh, *white bar*), as described for (**b**). All nocifensive behaviors were quantified from videos of the 5 min. immediately following subplantar injections. Injection regimens occurred on different days and opposite paws of the same mice. *Error bars* are SE. (***p > 0.001, **p > 0.01, *p > 0.05)

ANO1 channels are involved in nocifensive responses to a variety of noxious stimuli. Nocifensive behavioral responses to noxious heat, bradykinin and formalin have been significantly reduced in mice that lack functional ANO1 in their somatosensory neurons [6–9]. Capsaicin activation of somatosensory neuronal TRPV1 induces painful sensations [27]. In the prior section, we demonstrated that pharmacological inhibition of primary cultured DRG neuronal ANO1 diminished the frequencies of APs induced by capsaicin, in vitro. In this section, we examined if pharmacological inhibition of ANO1 could attenuate capsaicin-induced nocifensive behaviors in vivo. As expected, subplantar 25 μl injections of capsaicin (50 µM) into mice hind paws induced vigorous nocifensive behaviors of licking, flicking and lifting. The nocifensive behavioral responses to capsaicin were significantly less in number and time spent performing when the injected solution of 50 µM capsaicin was mixed with 1.3 mM ANO1-inh (following a pretreatment inject of the paws with 1.3 mM ANO1-inh 2 h earlier) (Fig. 5a, b). Time spent in nocifensive behavioral responses were further quantified by minutes following injection of capsaicin or ANO1-inh co-application/pre-treatment (Fig. 5c). *{Average total behaviors induced by: capsaicin (number* = *50.3* ± *2.9 and time spent* = *164* ± *12.5); capsaicin with ANO1*-*inh (number* = *30.6* ± *1.3 and time spent* = *60.5* ± *4.3); n* = *8}.* Vehicle (control) pre-treatment of the paws induced a significant increase (p < 0.05) in nocifensive responses to a following injection of 50 µM capsaicin {*Average total of behaviors (number* = *60.3* ± *2.8; time spent* = *236* ± *17.2); n* = *6*} than naïve paws injected with capsaicin. The data suggests that pain evoked by TRPV1 activation can be reduced by inhibiting ANO1. Thus, the data indicate ANO1 are involved in facilitating TRPV1 actions to evoke pain and inhibition of ANO1 could evoke analgesia.

## Discussion

Management of chronic peripheral pain is a major medical issue. In the last decades, great strides have been made in understanding the molecular mechanisms involved in pain generation and perception, and new treatments have resulted. Key cellular components of peripheral pain generation are nociceptors (somatosensory neurons). Noxicants and mediators of tissue inflammation and injury evoke action potentials (APs) that send signals to the spine and brainstem for eventual pain perception by the brain [4]. Nociceptors are ideal targets for treatments of pain. However, the molecular biology of nociceptors, and thus potential target sites, are not well defined. Recently, sensory neuronal Anoctamin 1 Ca^2+^–gated Cl^−^ channels (ANO1) have been demonstrated to transduce signals for the generation of peripheral pain in response to environmental and inflammatory signals [6–8, 32]. In sensory neurons, ANO1 and other Cl^−^ channels are excitatory due to the relatively high levels of intracellular Cl^−^ ([Cl^−^]_i_) [18]. ANO1 channels are activated by intracellular Ca^2+^ ([Ca^2+^]_i_), heat and pharmacological reagents, such as an *N*-aroylaminothiazole, E-act [17, 18]. We explored direct activation of ANO1 by E-act to strengthen the evidence that activation of sensory neuronal ANO1 Cl^−^ channels are an important molecular mechanism for the generation of peripheral pain. We found E-act triggers APs in sensory neurons. Interestingly, E-act effects were dependent upon [Cl^−^]_i_ but independent of heat or [Ca^2+^]_i_. Furthermore, E-act induces vigorous pain-associated nocifensive behaviors by mice in vivo.

Sensory neuronal [Cl^−^]_i_ is approximately 40 mM; this anion concentration determines that ANO1 and other Cl^−^ channels have a reversal potential (Cl^−^ electrochemical equilibrium (E_Cl-_)) near −30 mV [18]. Therefore, sensory neuronal ANO1 carries depolarizing Cl^−^ effluxes that could activate voltage-gated Na^+^ channels responsible for AP propagation. However, we repeatedly observed that sensory neuronal ANO1, activated by E-act, have outwardly rectifying current–voltage curves, similar to current–voltage curves recorded of native ANO1 in other tissues [6, 33]. This would imply that ANO1 carries relatively small currents associated with Cl^−^ effluxes. Possibly, currents of other channels, voltage regulation of ANO1, [Ca^2+^]_i_ modulation of ANO1 currents or other factors, are masking ANO1 carried inward currents (Cl^−^ effluxes) in the sensory neuronal current–voltage curves. In our studies, E-act-induced sensory neuronal ANO1 carried Cl^−^ effluxes (inward currents) that were large enough to depolarize the membrane to activate voltage-gated Na^+^ channels. E-act-induced APs were dependent upon [Cl^−^]_i_ being 40 mM or higher, which indicates Cl^−^ channel involvement. Studies of membrane receptor-activated sensory neuronal ANO1 (via [Ca^2+^]_i_) have reported larger inward currents carried by ANO1 (>400 pA), when the membrane was held at −60 mV [7, 32]. Inward current sizes of ANO1 are known to vary depending upon [Ca^2+^]_i_ levels in recombinant models [38]. ANO1 outward rectifying currents would also imply that ANO1 carry large influxes of Cl^−^ once APs are activated. Thus, ANO1 could enhance the repolarization phase of APs at depolarizing potentials. When we compared individual APs spontaneously occurring at baseline and APs induced by E-act application in the same DRG neurons, the APs were similar. However, a complete biophysical investigation of ANO1 channel carried ions in the initiation and repolarization of APs is needed, especially at different [Cl^−^]_i_.

Sensory neuronal ANO1 channels are involved in the physiology of acute peripheral pain responses to heat and inflammatory signaling. ANO1 channels are one of several thermosensitive ion channels in sensory neurons that detect harmfully high temperatures and subsequently evoke APs to signal for painful ‘hot’-associated nocifensive responses [8]. Sensory neuronal B_2_ and PAR-2 GPCRs detection of bradykinin and proteases released by injured or inflamed tissues results in an increase of [Ca^2+^]_i_ (via IP_3_ receptors) that activates ANO1. Thereby, ANO1 carry a depolarizing Cl^−^ flux that mediates these sensory neuronal GPCRs firing of APs and subsequent nocifensive responses at the site of injury [6, 7]. Conceivably, sensory neuronal ANO1 gating by raised [Ca^2+^]_i_, the unknown mechanisms of the intrinsic thermosensor or the unknown mechanisms of E-act, could be involved in various physiological and pathological conditions of heightened and chronic peripheral pain. Targeting sensory neuronal ANO1 with pharmacological antagonists could diminish peripheral pain of these conditions. Nocifensive behavioral responses to noxious heat, bradykinin and formalin have been shown to be significantly reduced in mice with functional ablation of ANO1 in their sensory neurons [8, 9]. We find that pharmacological inhibition of ANO1 suppresses E-act induced pain-associated nocifensive behaviors. Moreover, we find that an ANO1 inhibitor attenuates the triggering of APs and nocifensive behavioral responses induced by the sensory neuronal, noxious heat sensor, TRPV1.

TRPV1, TRPV4, TRPA1 and several other TRP channels are expressed in sensory neurons and are involved in somatosensory detection and nocifensive responses to environmental temperatures and reactive chemicals [22, 40, 42]. Recently, TRPV1 was shown to interact with ANO1 in cell membranes and carries Ca^2+^ into cells that thereby activates ANO1 in sensory neurons and co-transfected HEK-293 cells [32]. We and others postulate that TRPV1 carried [Ca^2+^]_i_-activates ANO1 [32]. The combined cation influxes carried by TRPV1 and anion effluxes carried by ANO1 would increase the size and speed of membrane depolarization and thus enhance the triggering of action potentials and subsequent pain perception [43]. Simultaneous with another laboratory’s findings, this hypothesis was tested and supported by our demonstration that blocking ANO1 channels will attenuate TRPV1 activator, capsaicin, induced firing of sensory neuronal APs and mouse nocifensive behavioral responses associated with pain [25–28, 32]. Similar to TRPV1, ANO1 could facilitate other sensory neuron TRP channels. TRPV4 carried [Ca^2+^]_i_ activation of ANO1 occurs in epithelia cells of the choroid plexus and in HEK-293 cells co-transfected with TRPV4 and ANO1, but has not yet been examined in neurons [33]. Sensory neuronal TRPA1 responds to reactive chemicals, such as formalin [4, 44]. Interestingly, formalin-induced nociception also involves ANO1 and TRPV4 [45, 46]. Perhaps nociceptor neuron depolarization by formalin activation of TRPA1 or TRPV4 is amplified by ANO1.

The mechanisms by which ANO1 interact with changes of [Ca^2+^]_i_ generated by membrane receptors in sensory neurons are controversial [7, 31]. Evidence suggests that sensory neuronal ANO1 are directly coupled with IP_3_ receptor complexes to provide Ca^2+^ in close proximity to gate ANO1 and to protect against cytosolic [Ca^2+^]_i_ from enhancing ANO1 sensitivity to heat or activating ANO1 to consequently produce pain [7]. This could explain why DRG neuronal ANO1 do not appear to be activated by increased [Ca^2+^]_i_ from voltage-gated Ca^2+^ channels [7]. TRPV1 carried [Ca^2+^]_i_ activates phospholipase Cδ (PLCδ). PLCδ cleaves IP_3_ to open IP_3_ receptor channels and thus could be the intermediator in TRPV1 activation of ANO1 [47, 48]. An alternative explanation is that TRPV1 direct coupling with ANO1 allows for TRPV1 channels to deliver large [Ca^2+^]_i_ in close proximity to activate ANO1 independent of IP_3_ receptors [32].

In addition to proteases and bradykinin, various other factors serve as signals of tissue damage and inflammation. The ‘inflammatory soup’ of factors (neurotropins, prostaglandins, ATP, serotonin, histamine, thymic stromal lymphopoietin, other cytokines…) activate metabotropic receptors and ion channels on adjacent sensory neuronal processes that increase [Ca^2+^]_i_ and trigger APs, resulting in pain, pruritus and nocifensive responses to the injured, inflamed area [49–51]. In other cell types, inflammatory factors activate ANO1 channels by Ca^2+^ release via IP_3_ receptor in a manner similar to PAR2 and B_2_ GPCRs [43, 52]. ANO1 could be involved in the various inflammatory factors activation of nociceptor neurons and transduction of pain. Thus, pharmacological antagonists of ANO1 could attenuate a variety of the peripheral mechanisms of acute inflammatory pain; in addition to attenuating the acute pain induced by TRPV1 or the detection of environmental hazards by other TRP channels.

Recent evidence suggests that sensory neuronal ANO1 Cl^−^ channels are involved in hyperalgesia and allodynia from tissue inflammation and peripheral neuropathy. Sensory neurons innervating inflamed tissues become hyperexcitable as a result of continuous exposure to ‘inflammatory soup’ signaling factors. These hyperexcitable sensory neurons contribute to the hyperalgesia and allodynia from inflammation. Genetic ablation of functional ANO1 in mouse sensory neurons results in diminished hyperexcitablity of sensory neurons incubated with ‘inflammatory soup’ factors. Furthermore, hyperalgesia and allodynia-like responses to heat and mechanical pressure in inflamed mouse paws and in paws with a damaged sciatic nerve are diminished for mice with ablation of functional ANO1 in sensory neurons [9]. Continuous exposure to ‘inflammatory soup’ signaling factors alters sensory neuronal intracellular Cl^−^ homeostasis resulting in continuously high [Cl^−^]_i_, which has a role in hyperexcitability [53, 54]. We hypothesize that ANO1 Cl^−^ channels’ contribution to inflammatory hyperalgesia involves the high [Cl^−^]_i_. The positive shift of E_Cl-_ due to the heightened [Cl^−^]_i_ levels would shift ANO1 channels’ Cl^−^ effluxes to readily occur at more depolarized potentials and with larger current sizes at negative potentials [40]. Thus, APs and pain perception triggered by noxious stimuli activation of sensory neuronal TRPV1 channels, bradykinin receptors and other receptors would be amplified in inflammatory conditions by co-activated ANO1 channels carrying larger depolarizing Cl^−^ effluxes. However, in our studies, high [Cl^−^]_i_ (160 mM) did not alter sensory neuron responses to capsaicin or E-act compared to physiological [Cl^−^]_i_ (40 mM). Possibly at lower doses of capsaicin or E-act, differences between high and physiological [Cl^−^]_i_ modulation of sensory AP firing might be observed. In the opposite scenario, we observed that ANO1 channels inhibited AP firing by E-act or capsaicin of sensory neurons with low [Cl^−^]_i_ (10 mM). This phenomenon is similar to fetal brain neuronal Cl^−^ channels, which trigger APs due to high [Cl^−^]_i_, in contrast adult brain neuronal Cl^−^ channels inhibit AP generation due to low [Cl^−^]_i_ [54]. These findings support evidence that lowering sensory neuron [Cl^−^]_i_ favors analgesia and suggests a role of ANO1 in the increased [Cl^−^]_i_ levels that evoke inflammatory hyperalgesia [53, 54].

## Conclusion

In summary, our results provide strong evidence of a role for somatosensory neuronal ANO1 Ca^2+^-gated Cl^−^ channels in the molecular mechanisms of nociception transduction. We report that: (1) direct pharmacological activation of ANO1 channels induces [Cl^−^]_i_-dependent AP firing in DRG neurons and (2) this is translated into ANO1 activation that evokes nocifensive behaviors associated with pain. Furthermore, we provide indirect evidence that ANO1 channels are involved in TRPV1-induced firing of APs and nocifensive behavioral responses. We demonstrated that: (1) pharmacological inhibition of ANO1 attenuates AP firing by DRG neurons from TRPV1 activation by capsaicin (dependent upon [Cl^−^]_i_) and (2) co-treatment with an ANO1 inhibitor alleviated capsaicin-induced nocifensive behavioral responses by mice.

Billions of dollars are spent on prescribed and over-the-counter medications to alleviate peripheral pain [2]. We show that pharmacological inhibition of ANO1 attenuates capsaicin-induced nocifensive responses in a mouse model of peripheral pain. Others have shown that pharmacological inhibition or genetic ablation of sensory neuronal ANO1 attenuates mouse nocifensive responses to bradykinin, formalin and heat, as well as diminishing inflammatory hyperalgesia and neuropathic hypersensitivity [6, 8, 9]. Most likely ANO1 are involved in the nociception to a variety of noxious stimuli and pathological conditions. Therefore, inhibiting ANO1 channels have great potential as a new target for analgesics.

## Methods

### Animals

Female, 12–20 week old, Balb/c mice were housed at AALAC-accredited facility in standard environmental conditions (food/water ad libitum, 12 h light/dark light cycle, RT 24 °C). Animals were habituated before testing of in vivo experiments. All animal procedures were approved by the IACUC committee of Texas A&M Health Science Center-Institute of Biotechnology.

### Dorsal root ganglia harvest

Adult mouse dorsal root ganglia (DRG) somatosensory neurons were dissected immediately following death by CO_2_ euthanasia and dissociated by 1 h incubation in 0.26 WU/ml Liberase TM (Roche), followed by washes with Hank’s solution, trituration, and straining of debris (40 μM Cell Strainer; BD Falcon). Neurons were cultured in neurobasal-A medium (Invitrogen) with B-27 supplement, 0.5 mM glutamine, and 50 ng/ml nerve growth factor (Calbiochem) on 35 mm cell culture dishes coated with poly-d-lysine (Sigma-Aldrich) and laminin (Invitrogen). Cells were placed in an incubator in 95 % air/5 % CO_2_ atmosphere at 37 °C [56].

### Cell-line Culture and Transfection

HEK-293t cells were grown in Dulbecco’s modified Eagle medium (Gibco) with 10 % fetal bovine serum and 1 % penicillin/streptomycin at 37 °C in 5 % CO_2_. Cells were transiently transfected using Lipofectamine (Invitrogen) with 500 ng cDNA encoding mouse ANO1/TMEM16A in a mammalian expression vector fused with pEGFP fluorescent reporter [10]. Mouse ANO1/TMEM16A cDNA and plasmid was a generous gift from L. Jan laboratory (UCSF, San Francisco, CA). Transfected cells were incubated at 30–50 % confluency on poly-d-lysine coated glass coverslips overnight and identified for electrophysiology by GFP fluorescence using FITC filter (31001, Chroma).

### Electrophysiology

The day following (16–26 h) DRG dissection or transfection of cells, patch clamp electrophysiological experiments were performed in the perforated-patch (amphotericin-B, 250 μg/ml; MP Biomedicals) or whole-cell configurations with borosilicate micropipettes (GC150T, Harvard Apparatus) of resistances 2–5 MΩ pulled by PMP102 apparatus (Micro Data Instruments). EPC-10 Plus amplifier coupled with an analogue–digital converter (HEKA, Germany) recorded and manipulated voltages that were interpreted by PatchMaster software (V2X60). Bath solution contained (in mM): 140 NaCl, 5 KCl, 2 MgCl_2_, 2 CaCl_2_, 10 Hepes and 5 glucose (pH 7.3). A perfusion system exchanged prior bath solutions to bath solutions containing: 10 µM E-act (3,4,5-trimethoxy-*N*-(2-methoxyethyl)-*N*-(4-phenylthiazol-2-yl)benzamide; EMD Chemicals [18]) or 15 µM capsaicin (Enzo) with or without 20 µM ANO1-inh (T16A[inh]-A01; 2-[(5-ethyl-1,6-dihydro-4-methyl-6-oxo-2-pyrimidinyl)thio]-*N*-[4-(4-methoxyphenyl)-2-thiazolyl] acetamide; EMD Chemicals [34]). In certain experiments 10 µM ruthenium red (Enzo) was also in the perfused bath solution. Liquid junction potentials were calculated by Igor Pro software Patcher’s Power Tools (Wavemetrics) and adjusted for in PatchMaster.

Voltage-clamp high resolution currents were filtered at 2.3 kHz and digitalized at 100 µs intervals with holding potential of −70 mV or 0 mV, at every second voltage-ramps of −100 to +100 mV over 200 ms occurred as described previously [55]. Recording pipette solution contained (in mM): 150 CsCl, 2 MgCl_2_, 2.3 Na_3_ATP, 1.1 EGTA, 10 Hepes; pH 7.2. In certain experiments, free [Ca^2+^] was not 0, but was adjusted to 1 µM or 10 µM with addition of 1 mM or 1.12 mM CaCl_2_ as determined by Webmaxc, http://www.stanford.edu/~cpatton/webmaxcS.htm.

Current-clamp voltages were recorded using either amphotericin B-perforated patch (250 µg/ml) or whole cell configuration [6]. Initially, voltage-clamp was established as described above. If voltage-gated channels were noticeable, then current-clamp was begun. Using voltage ramps of −100 to +100 mV, the following reversal potentials were determined for the experimental groups: extracellular capsaicin (perforated-patch) with High [Cl^−^]_i_ at −42 ± 3 mV, Mid [Cl^−^]_i_ at −51 ± 9 mV and Low [Cl^−^]_i_ at −41 ± 6 mV; extracellular E-act (whole-cell) with High [Cl^−^]_i_ at -60 ± 2 mV, Mid [Cl^−^]_i_ at −28 ± 5 mV and Low [Cl^−^]_i_ at -54 ± 2 mV. Before beginning membrane voltage potential (V_m_) recordings, empirical measurements identified current injection sizes required to bring V_m_ to −70 ± 10 mV (to “reset” voltage-gated channels) and to −30 ± 10 mV (just below the voltage required to activate voltage-gated channels) for that specific DRG neuron. There appeared to be a technical issue in our current clamp that up to −100 pA current injection was necessary to lower V_m_ to −70 mV and approximately 0 pA injection for V_m_ of −30 mV. This could be due to faulty-sealed “leaky” patches in those patches with reversal potential near -30 mV. A sequence of square pulses of current injections with the following current injection protocol were employed to record V_m_: *1) [*−*40 to* −*100] pA for 400* *ms (to hold V*_*m*_*near* −*70* *mV); 2) 0* ± *10 pA for 400* *ms (to bring V*_*m*_*near* −*30* *mV); 3) [*−*40 to* −*100] pA for 20* *ms; 4) 0* ± *10 pA for 120* *ms; and 5) (*−*40 to* −*100 pA) for 100* *ms* [40] (see Fig. 2a). Action potentials (APs) only occurred during the 0-pA current injection segments of the protocol. Perplexing to the authors was that, at most, only one AP was observed for a 0-pA current injection segment and not a series/“train” of APs. The sequence of current injections was identical in perforated patch current-clamp voltage recordings. Only V_m_ “spikes” above 10 mV were considered as APs. Recording pipette solution for high [Cl^−^]_i_ condition contained (in mM): 150 KCl, 5 MgCl_2_, 10 HEPES, pH 7.4. Low [Cl^−^]_i_ condition solutions were identical, except KCl was replaced by equimolar K-gluconate. Mid [Cl^−^]_i_ condition solutions contained (in mM): 30 KCl, 110 K-gluconate, 5 MgCl_2_, 10 HEPES.

### Nociceptive behavior

Mouse hind paws received intraplantar injections of 25 μl of pharmacological reagent or vehicle using a 50 μl Hamilton syringe and 30G needle. Both experimental and control conditions were investigated within the same mice. To control for injection sequence, half of the mice in the experimental group received the hind paw injection(s) for one condition, while the other half received hind paw injection(s) of a different condition. The next day, mice received in the opposite hind paw the injection(s) of the condition that they had not yet received. The vehicle for all solutions was (v/v) 95 % propylene glycol and 5 % DMSO. In one experiment, mice were injected with vehicle in the left hind paw and ANO1 activator (5 mM E-act) in the right hind paw, in the manner just described. In a separate experiment (with separate mice), mice were injected with 5 mM E-act in the right hind paw and ANO1 inhibitor (1.3 mM T16A[inh]-A01; ANO1-inh) with 5 mM E-act in the left hind paw. In capsaicin experiments (again with separate mice), mice received an initial injection in the right hind paw of ANO1 inhibitor (1.3 mM T16A[inh]-A01; ANO1-inh) followed 2 h later with an injection of a mixture of 1.3 mM ANO1-inh with 50 µM capsaicin in the same paw or they received an injection of just 50 µM capsaicin in the left paw. As a further experimental control (with separate mice), 50 µM capsaicin paw injections were given in paws injected with vehicle 2 h earlier. Mice were placed in a Plexiglas enclosure and videotaped for 5 min immediately following final hind paw injections. Nocifensive behavior responses (paw flicking, lifting, and licking) were quantified by slowing the video frame speed with Windows Media player software [57].

### Statistics

For all statistical tests, a *p* value less than 0.05 was considered significant. Statistical analysis and graphic display of electrophysiological records were made with Igor Pro (Wavemetrics). Statistical errors were the standard error (SE). Average total action potential propagations were assessed by a one-way repeated measures ANOVA, followed by post hoc analysis with Bonferroni multicomparison test (SPSS). Dependent (repeated-measure) Student’s *t* were performed on average time of AP voltage peak and the number and time (either over a min or 5 min) spent flicking, lifting, licking and total nociception responses to injections of: (1) vehicle compared to ANO1 activator E-act, (2) E-act compared to ANO1 inhibitor T16A[inh]-A01 with E-act, and (3) capsaicin compared to capsaicin with T16A[inh]-A01.

## Additional file

**Additional file 1: Figure S1.** Recombinant mouse ANO1 currents induced by 10μM-[Ca2+]i and diminished by an ANO1-inhibitor T16A[inh]−A01. Traces of currents recorded to voltages ramped from −100 to +100mV activation by 10μM [Ca2+]i (blue trace) and suppression by ANO1-inhibitor, 20μM T16A[inh]−A01 (ANO1-inh, green trace) in a representative mANO1 transfected HEK−293t cell; VH=0mV. Individual traces of currents recorded at ─80mV and +80mV with 10μM-[Ca2+]i and inhibited by 20 μM Ano1-inh bath perfusion of mANO1 transfected HEK−293t cells (n = 4).

## Abbreviations

ANO1: anoctamin-1 channels
TRP: transient receptor potential
TRPA1: TRP ankyrin 1
TRPV1: TRP vallinoid 1
TRPV4: TRP vallinoid 4
BK: bradykinin
IP_3_: inositol-triphosphate
PAR2: proteinase activated receptor-2
GPCR: G-protein coupled receptor
E-act: *N*-aroylaminothiazole ANO1-activator
ANO1-inh: T16A[inh]-A01
min: minute
s: second
ms: millisecond
mANO1: recombinant mouse ANO1
mV: millivolts
pA: picoamperes
nA: nanoamperes
V_m_: membrane potential
DRG: dorsal root ganglia
HEK-293t: human embryonic kidney-293t
“I-V curve”: currents to voltage curve
AP: action potential
ATP: adenosine triphosphate
