# Supplementary figures and images for: Anoctamin-1 Cl− channels in nociception: activation by an N-aroylaminothiazole and capsaicin and inhibition by T16A[inh]-A01
Source: Mol Pain. 2015 Sep 12;11:55. doi: 10.1186/s12990-015-0061-y (PMC4567824; doi:10.1186/s12990-015-0061-y)

Supplementary Figure 1S

HEK-293 mANO1 10  $\mu$ M  $[Ca^{2+}]_i$

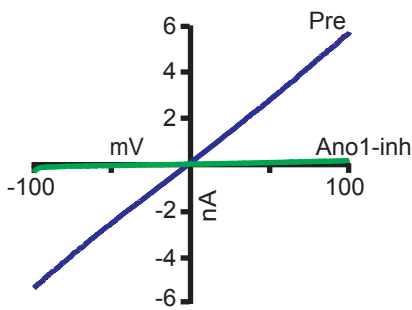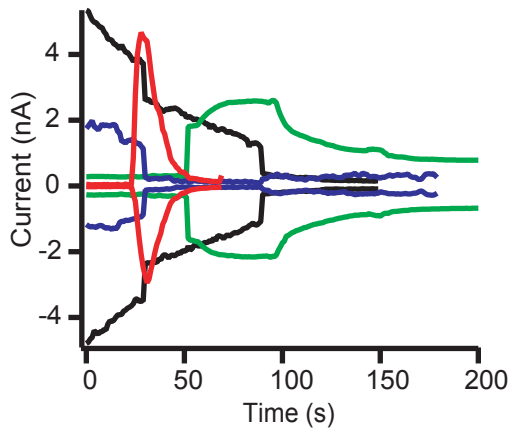

Supplement: Supplementary file 1 — Additional file 1: Figure S1. Recombinant mouse ANO1 currents induced by 10μM-[Ca2+]i and diminished by an ANO1-inhibitor T16A[inh]−A01. Traces of currents recorded to voltages ramped from −100 to +100mV activation by 10μM [Ca2+]i (blue trace) and suppression by ANO1-inhibitor, 20μM T16A[inh]−A01 (ANO1-inh, green trace) in a representative mANO1 transfected HEK−293t cell; VH=0mV. Individual traces of currents recorded at ─80mV and +80mV with 10μM-[Ca2+]i and inhibited by 20 μM Ano1-inh bath perfusion of mANO1 transfected HEK−293t cells (n = 4). [file 12990_2015_61_MOESM1_ESM.pdf]
